# The Role of Corticosteroids in Adult Respiratory Distress Syndrome caused by Viridans Group *Streptococci* Bacteremia in Neutropenic Patients

**DOI:** 10.4084/MJHID.2014.055

**Published:** 2014-09-01

**Authors:** Abraham T. Yacoub, Lysenia Mojica, Lily Jones, Andrea Knab, Sally Alrabaa, John Greene

**Affiliations:** 1 H. Lee Moffitt Cancer Center and Research Institute. 12902 Magnolia Drive, Tampa, Florida 33612-9497; 2 University of South Florida, Division of Infectious Diseases. 1 Tampa General Circle, G323, Tampa, FL 33606

## Abstract

In the past decades, viridans group S*treptococci* (VGS) have emerged as an important cause of bacteremia in neutropenic patients with cancer. The clinical course of VGS bacteremia can be devastating including septic shock and adult respiratory distress syndrome (ARDS). It has been suggested that septicemia with VGS triggers the development of noncardiogenic pulmonary edema in patients with pre-existing damage of the lungs due to aggressive cytotoxic treatment. Thus, the preemptive administration of corticosteroid to patients diagnosed with VGS bacteremia with early onset of respiratory failure has been employed to prevent ARDS. While this management strategy has been suggested in the literature, little published data are available to validate this practice. In this study, we sought to review the benefit of early administration of corticosteroid to patients who developed symptom or early signs of respiratory failure while being neutropenic with VGS bacteremia.

## Introduction

During the last decades, gram-positive bacteremia has increased dramatically. Gram-positive cocci are the most frequent cause of nosocomial bloodstream infections. Among Gram-positive cocci, Viridans streptococci are a common cause of bacteremia in cancer patients with neutropenia, causing serious complications such as pneumonia, septic shock, and ARDS.^1^–^6^ We present a series of cases of VGS bacteremia complicated with ARDS; early initiation of corticosteroids resulted in complete recovery.

## Materials and Methods

A retrospective chart review of patients with hematologic malignancy diagnosed with VGS bacteremia admitted to the Moffitt Cancer Center in Tampa, Florida between 1/1/2001 and 4/1/2012 was completed. Data was collected about respiratory symptoms, diagnosis of adult respiratory syndrome, results of blood cultures, medications received and outcome. Sepsis was defined as suspected or microbiologically proven infection together with Systemic inflammatory response system (SIRS). The SIRS criteria was defined as temperature less then or equal to 36 ° C or greater then or equal to 38 ° C. Heart rate greater then or equal to 90. Respiratory rate greater then 20 breaths per minute or a partial pressure of carbon dioxide (PaCO2) less then 32 mmHg and a white blood cell (WBC) count greater then or equal to 12,000 cell/mm3 or less then or equal to 4,000 cell/mm3 or greater then 10% bands. VGS bacteremia was defined as growth of viridans streptococci from at least one peripheral or central blood sample. Neutropenia was defined as having an ANC lower than 500/μL

## Results

In this study, 70 cases of VGS bacteremia in neutropenic patients were reviewed. The most common adverse event of VGS bacteremia in this group of patients is the development of serious pulmonary complications such as ARDS. In our study (Table 1), 7 patients developed ARDS. The ages of all 7 patients ranged from 22 to 75. There were 4 females and 3 males. There were 3 Caucasian patients, 1 African American patient, 1 Hispanic patient, and 1 Middle Eastern patient. Acute lymphoblastic leukemia was found in 4 patients and Acute myelogenous leukemia was found in 3 patients. The type of chemotherapy for the management of the hematological malignancies was not available. All of the patients were neutropenic. The average length of neutropenia was 11 days. The average of the length of stay in the hospital was 35 days. The most common identifies streptococcal species were S*treptococcus mitis and Streptococcus oralis,* isolated in 4 of 7 patients. The most common antibiotics administered after positive blood cultures were cefepime, vancomycin, and meropenem. All 7 patients received corticosteroids early with the onset of respiratory failure. The most commonly prescribed regimen was methylprednisolone 60 mg intravenously every 12 hour for an average of 3 days. All patients received comparable supportive care, appropriate antibiotics, ventilation and hemodynamic support. All patients (100 %) recovered from respiratory failure after receiving corticosteroids. One patient expired due to Graft-versus-Host Disease complication post chemotherapy treatment. There were no significant adverse events attributable to steroids use.

## Discussion

Viridans streptococci are gram-positive spherical bacteria that characteristically form pairs or chains during growth. They are catalase-negative, facultative anaerobic, non-motile, and do not produce spores or gas.^5^

The viridans streptococci include *S mitis, S mutans, S salivarius, S sanguis,* and others. Viridans streptococci typically are alpha-hemolytic, optochin-resistant, their colonies are not soluble in bile, and they have carbohydrate fermentation patterns.^7^ They are the most prevalent members of the oral normal flora, other areas of the upper respiratory tract, and are important for the healthy state of the mucous membranes there. They also commonly inhabit the gastrointestinal and female genital tracts.^7^,^8^ The viridians streptococci are described as organisms of low virulence, however these organisms may invade sterile body sites, which can lead to life-threatening diseases (e.g., endocarditis, meningitis, and pneumonia).^5^–^7^

Viridans streptococcal bacteremia occurs frequently in neutropenic patients, who have impaired host defense mechanisms and especially when the oral mucosa is disrupted, causing serious complications. Serious complications described include: a shock syndrome, characterized by hypotension, rash, palmar desquamation, and adult respiratory distress syndrome.^2^,^3^,^5^,^6^ ^16^,^20^ The mortality rate among patients with viridans streptococcal bacteremia who develop complications is high, up to 80% in some case series.^2^,^3^,^6^ *Streptococcus mitis* is the species most frequently isolated from the patients who have developed serious complications from viridans bacteremia like sepsis and/or adult respiratory distress syndrome (ARDS).^3^,^6^,^9^,^10^,^17^,^18^,^19^

The incidence of viridans streptococcal bacteremia has increased during the last decades.^2^,^3^ In a study conducted in a university hospital for adults in Barcelona, Spain, of 485 episodes of bacteremia in neutropenic patients, viridans streptococci caused a total of 88 episodes (18%), and 10 of these patients developed serious complications such as ARDS and septic shock. From the 10 patients that developed complications, 7 involved S*treptococcus mitis*.^3^ These complications were associated with a high mortality rate (80%), secondary to the development of multi-organ failure.

The risk factors associated with viridans streptococcal bacteremia that have been identified are oral mucositis, profound neutropenia, high-dose chemotherapy like cytosine arabinoside, bone marrow transplantation, antimicrobial prophylaxis with trimethoprim-sulfamethoxazole or a fluoroquinolone, and the use of antacids, histamine type 2 receptor antagonists or proton pump inhibitors.^1^–^3^,^6^,^11^–^13^

Some studies propose that the administration of trimethoprim-sulfamethoxazole or fluoroquinolone, as well as the use of antacids to prevent chemotherapy induce gastritis may predispose to overgrowth of viridans streptococci in the gastrointestinal tract.^5^ One study found that the significant risk factors to developed serious complications were: severe oral mucositis, high-dose chemotherapy with cyclophosphamide, and allogenic bone marrow transplantation.^3^

The pathogenesis of these complications remains unknown. It is believed that neutrophils play a key role in the development of ARDS.^10^ There is evidence that viridans streptococci can induce proinflammatory cytokines including TNF-α, TNF-β, IL-6, and IL-8. The up-regulation of intercellular adhesion molecule (ICAM)-1 by *Streptococcus mitis* has also been demonstrated. Of note, IL-8 has an important association with lung damage in patients with ARDS. Viridans streptococci do not produce lipopolysaccharide (endotoxin), and there is no substantial information reported regarding the ability of these microorganisms to produce exotoxins. Therefore, it has been postulated that the pathogenesis of streptococcus viridans causing septic shock and/or ARDS is of host immune etiology.^5^,^9^,^10^,^11^,^14^,^15^ One study conducted in Germany, found much higher levels of IL-6 in 2 patients with lethal alpha-hemolytic streptococcus septic shock, than in controls with uncomplicated gram-positive bacteremia.^15^ Another study using enzyme-linked immunoabsorbent assays compared the ability of cell-free bacterial supernatants derived from commensal and clinical strains of viridans streptococci to induce pro-inflammatory cytokines. In their results they reported that supernatants from clinical isolates induced significantly more TNF-β, and IL-8 than did supernatants from commensal strains.^10^ Given the fact that IL-8 is a chemoattractant cytokine for neutrophils, and neutrophils are involved in the pathogenesis of ARDS, some studies have proposed the association between the increased levels of IL-8 in viridans streptococcal bacteremia and the development of ARDS.^10^ One study found that of 52 viridans streptococcus strains isolated from the blood of neutropenic patients, all induced the production of TNF-α, IL-1β, and IL-8. This study also suggested the ability of viridans streptococcus to cause the up-regulation of ICAM-1 and subsequent development of shock.^9^

The administration of moderate dose corticosteroids may be beneficial in preventing the development of ARDS in patients with *Streptococcus mitis* bacteremia. Our findings reflect 100% recovery from a complication (ARDS) that carries between 30–80% mortality. Furthermore, moderate doses of steroids with short duration of administration were not associated with significant adverse events in our case series. At University Hospital St. Radboud, Nijmegen at The Netherlands, 11 patients with *Streptococcus mitis* bacteremia following chemotherapy received high doses of corticosteroids pre-emptively. None of them developed ARDS.^14^ These findings and our observation of improved mortality with early administration of corticosteroids with Streptococcus viridans bacteremia in neutropenic cancer patients who develop respiratory complications, are of great clinical interest. Further studies are warranted.

## Conclusion

*Streptococcus mitis* is the species most frequently isolated from the patients who have developed ARDS from Streptococci viridans group bacteremia. Our data suggest that the early administration of corticosteroids to neutropenic patients who develop early signs of respiratory failure with VGS bacteremia can prevent the progression of ARDS and improve survival. Moderate doses of steroids with short duration of administration were not associated with significant adverse events in our case series. While the use of corticosteroids in this setting has been described in the literature since the early 1990s, there remains a scarcity of data and our study help shed some light on this area. Moreover there is little recognition among clinicians of the association between ARDS and VGS bacteremia (particularly *mitis* species in neutropenic cancer patients) and thus this treatment modality is used late in the course of illness which may reduce benefit. Further studies are warranted to validate these findings and to further examine the utility of preemptive use of corticosteroids in cancer patients who develop VGS bacteremia, in regards to ARDS incidence reduction.

## Figures and Tables

**Table 1:** Adult Respiratory Distress Syndrome caused by Viridans Group *Streptococci* Bacteremia in Neutropenic Patients Treated with Corticosteroids.

Age	Gender	Malignancy	Days of hospital stay	Days of Neutropenia	Diagnosis	Microbiology	Treatment	Outcome
38	F	ALL	7	3	ARDS, pulmonary edema, bilateral infiltrates and pleural effusion	*Streptococcus viridans*	cefepime, vancomycin, meropenem, ceftriaxone, sulfamethoxazole/tri methoprim, and methylprednisolone 60 mg intravenous every 12 hour for 3 days.	Cured
26	M	ALL	40	5	hypoxia, diffuse ground glass opacities bilaterally, pulmonary hemorrhage, and pulmonary edema	*Streptococcus mitis*, and *Streptococcus oralis*	cefepime, daptomycin, meropenem, sulfamethoxazole/tri methoprim, and methylprednisolone 60 mg IV	Cured
75	F	AML	26	29	ARDS, ground-glass infiltrates, and shortness of breath	*Streptococcus mitis,* and *Streptococcus oralis*	cefepime, vancomycin, doxycyclin, and methylprednisolone 60mg IV	Cured
33	F	ALL	24	14	ARDS, diffuse alveolar hemorrhage, and bilateral ground-glass opacities	*Streptococcus mitis*, and *Streptococcus oralis*	zosyn, vancomycin, ceftriaxon, cefepime, doxycyclin, cephalexin, sulfamethoxazole/tri methoprim, and methylprednisolone 125 mg IV	Cured
36	M	AML relapsed	30	10	ARDS, ground-glass pnemonia, and septic shock	*Streptococcus mitis*, and *Streptococcus oralis*	cefepime, vancomycin, meropenem, daptomycin, linezolid, and methylprednisolone 60mg IV	Cured
22	F	ALL	99	5	ARDS, ground-glass pnemonia due to diffuse alveolar hemorrhage, and bilateral pulmonary infiltrates	*Streptococcus viridans*	vancomycin, cefepime, meropenem, sulfamethoxazole-trimethoprim, and methylprednisolone 60mg IV	Cured
37	M	AML	18	14	ARDS, ground-glass pneumonia	*Streptococcus viridans*	zosyn, daptomycin, meropenem, levofloxacin, vancomycin, and hydrocortisone 100 mg IV	Cured

AML indicates Acute myeloid leukemia; ALL, Acute lymphocytic leukemia; ARDS, Acute respiratory distress syndrome; IV, Intravenous
